# Epidemic Cholera: Its History, Causes, Pathology and Treatment

**Published:** 1849-05

**Authors:** C. B. Coventry

**Affiliations:** Prof, of Obstetrics and Medical Jurisprudence, in the Medical Institution of Geneva College. Prof, of Physiology and Medical Jurisprudence in the University of Buffalo


ART. VI.—*Epidemic Cholera: its History, Causes, Pathology and Treatment.* By C. B. COVENTRY, M. D., Prof. of Obstetrics and Medical Jurisprudence, in the Medical Institution of Geneva College. Prof. of Physiology and Medical Jurisprudence in the University of Buffalo.

"Internal sanatary arrangements, and not quarantine or sanatary lines, are the safeguards of nature against epidemic diseases."—FARR.—*Registrar General.*

Buffalo; Geo. H. Derby & Co. Publishers. 1849. 18mo. pp. 119.

This work was placed upon our table, just as the manuscript of the last form of the April number of this journal was about to be committed to the hands of the printer, and we were then only able to announce the fact of its publication. Justice to the work requires a more extended notice, after a careful examination of its contents. Prof. Coventry was induced to prepare this brief, but comprehensive monograph, in consequence of numerous applications for copies of his report on epidemic cholera, made to the Faculties of the medical colleges of Geneva and Buffalo, which was published in this Journal, volume IV, 1848. This report embodied the results of his investigations of the disease, during the former and late prevalence of the epidemic; having been deputed by the Common Council, and

Board of Health, of Utica, in 1831, to visit places which were then suffering from the disease, and, in 1847–8, visiting Europe under instructions from the Colleges of Geneva and Buffalo, to resume his investigations. In addition to these occasions and incentives for observation, the author had ample opportunity for becoming familiar with the phenomena of the affection, and of testing the practical applicability of his views, at the bedside, during the prevalence of the epidemic at the city of Utica, the place of his residence. The present volume, therefore, is not a mere compendium of facts and opinions entertained by the best authorities on the subject, but contains the fruits of his own deliberate examination and reflections, based upon considerable experience in the treatment of the disease. Hence, the practical views enunciated, have all the force to be derived from the author's well known ability as an accurate observer, a sound thinker, and judicious practitioner. As such, we cordially commend the work to our readers, and hope that it may have a circulation commensurate with its claims upon the attention of the profession.

The *history* of the disease occupies the first third of the volume. We pass by this portion, to the second subject, viz., the *etiology*, which, it is obvious, would be the most important subject of all, were we able to arrive at any precise results respecting the nature, source, diffusion, and action of the remote cause, or causes.

That our information on these points is infinitely less in amount than could be desired, and, in character, is inferential and hypothetical, rather than demonstrative, must be admitted; but this remark will equally apply to the etiological information we possess of many other diseases, especially epidemic diseases. Prof. C. attributes cholera to a special epidemic cause, without committing himself to any of the fanciful theories which have been advanced on this subject. But he attributes, also, no small importance to various collateral, or concurring causes, which he classifies under the name of *sporadic causes*, the coöperation of some of them being more or less involved in almost all cases. These causes are far more appreciable than the specific cause, and, moreover, may be, to a considerable extent, controlled; hence the vast importance of hygienic regulations, in arresting, or limiting the prevalence of this terrible malady.

Prof. C. is not an advocate of the doctrine of contagion. He is disposed to doubt the existence even of what has been called "Contingent Contagion." He would account for the occasional diffusion of the affection among inmates of hospitals, by the operation of the various circumstances appertaining to such institutions which are likely to act as cooperating, or exciting causes. He states that "all past experience goes to prove, that hospitals properly ventilated and cleansed, are no more liable to produce the disease than other places."

The subject of contagion has engrossed a considerable share of the literature of cholera, as indeed, of all epidemic affections. It had seemed, however, as the author remarks, to have been settled pretty conclusively' in so far as the general unanimity of the Profesion is concerned, albeit the doctrine has had several powerful advocates, among whom are Drs. Copeland and Watson of England. Facts connected with the recent prevalence of the disease at New York, New Orleans and other southern places, have appeared to favor the supposition of its contagiousness, and the number of those now disposed to favor its diffusion in this way is greater than formerly.

We are not prepared to deny *in toto* its personal communicability, but we think it can be established by a variety of considerations, rendering the position as strong as it could well be without demonstration, that the principle of contagion if it exists, is limited in its operation, and will by no means account for the diffusion of the disease. If it be communicable in any sense like the eruptive fevers, or even typhus, it certainly offers a striking contrast with all the laws respecting the operation of contagion in these affections.

The author next treats of the *symptoms* of cholera. He divides the disease into *four* stages. The *first* stage embraces what have been frequently called the premonitory symptoms. Prof. C. regards it as highly important to consider this as a veritable stage of the disease. He considers it to be entitled to be distinguished as the "curable stage," and thinks that at this period the disease may be arrested with almost certainty by judicious means. The *second* stage is marked by the rice water evacuations, cramps, coldness, feeble pulse, extreme thirst, &c. peculiar burning sensation at the pit of the stomach. The *third* stage is characterised by that array of symptoms constituting the *state of* collaps, with which the reader is sufficiently familiar. The *fourth* stage, which the author thinks embraces *sequences* of the disease, rather than what are properly its *symptoms*, consists of the phenomena supervening when reaction occurs after the state of collapse, phenomena denoting a condition almost perfectly analagous to typhoid fever. Patients often succumb in this conditon, after having passed through the preceding stages.

The *Pathology* is the subject which follows next in order. After reviewing briefly some of the views entertained by different authorities, the author expresses the conclusions to which he has been led by his own observations and reflections, as follows: —

"Whatever be the nature of the poison, and whether its primary action be on the blood, or the nervous system, the effect is evidently to prevent those changes in the circulation by which heat is generated, and the vital current purified. Carbonic acid is not formed by the union of the carbon with oxygen; heat is not generated; the blood, still charged with carbon, paralyzes the action of the heart, which, in its turn, becomes enfeebled; congestion of the internal organs follows, as in actual asphyxia; the nervous system being also paralyzed, the congested organs are incapable of retaining their contents, and the watery portion of the blood escaping by transudation into the intestinal canal, is ejected by vomiting and purging; hence, the discharge; hence, the discoloration from retention of the carbon; hence, the extreme cold, in consequence of heat not being generated, and of the rapid evaporation from the surface; hence, also, the dark color, and thickened consistency of the circulating fluid, and, hence, too, the reason why injection of saline fluids into the veins seldom succeeds in restoring the patient, though it may revive him for a time, for it cannot remove the congestion of the capillary system.*

"Dr. E. A. Parkes, of London, in a recent work on the subject, has advanced, and endeavored to maintain the doctrine, that the poison of the atmosphere acts primarily on the blood. I am, however, disposed to believe that the condition of the blood, upon which he founds his principal evidence, is not the primary affection, but that the impression is primarily on the nervous system, prostrating its energies, and thus preventing those changes in the condition of the blood, and nutritive processes, by which the blood is purified, and caloric evolved. Dr. Parkes thinks that the changes induced in the function of respiration, are directly consequent upon the alteration of the blood, and are the proper and distinctive symptoms of the disease. We suppose, on the contrary, that the changes in the function of respiration are induced by deficiency in nervous energy, and that the altered state of the blood, follows as an effect, instead of being the cause."

We come now to that most important of all subjects connected with the disease, viz., its *treatment.* To the remarks under this head it would be impossible to do proper justice without quoting them entire. We shall briefly state some of the more important points, refering the reader, as also with respect to the topics embraced in the other divisions, to the work itself, for a fair exposition of the author's opinions. We have no specific remedies for cholera, consequently the appropriate therapeutical measures must be tried upon rational principles, and the results of experience, and the former will, of course, bear a close relationship to the views entertained respecting the pathology.

---

* Nearly all writers and observers agree that congestion of the internal organs, and of the venous system in particular, is one of the most constant and striking of the phenomena in cholera. We fear, however, that sufficient discrimination has not been used, as to the important pathological distinction between active and passive congestion; for the first, blood-letting is an almost certain remedy, whilst, for the second, its utility is frequently more than problematical. No doubt the congestion in cholera, is of the latter character, and unless we can secure some evidence of reaction, there would be no certainty of the congestion being relieved by bleeding.

Prof. C. submits the treatment adapted to each stage under a distinct head. The objects to be attained by remedies in the *first* stage are, to "remove nervous prostration, and the congestion which has already commenced, by equalising the circulation." For these objects, are recommended confinement to bed and warm mild aromatic drinks, with a view to procure perspiration; this should be promoted by Dover's powder, in combination with calomel in small quantities; or, by a combination of opium, camphor and calomel, as follows: — opium gr. $\frac{1}{4}$, camphor gr. ss., calomel gr. i, repeated every two hours. A small dose of rheubarb and magnesia' or pure castor oil, should be given after five or six doses of the above have been administered.

An emetic of ipecacuanha, or sulph. zinc, may be administered, if there is a sense of nausea and oppression, or if the stomach is oppressed by a recent meal. Bleeding, carefully practiced, watching the effect, is called for if the patient be of a full, strong, or plethoric habit. Great care to prevent relapses should be observed for several days after the symptoms of this stage are relieved. The vast importance of resorting to judicious treatment in this stage is enforced, and the author, as already stated, expresses a confident belief, that, if appropriately treated in this stage, the disease may, with almost certainty, be arrested.

Owing to the fact that the symptoms of the *first* stage very often do not excite sufficient alarm, or attention, to lead the patient to apply personally for medical aid, a large proportion of the cases which come under treatmet present the phenomena of the second stage fully developed. The treatment recomended by the author in this stage, is too succinctly expressed to admit of condensation, and we therefore give it in his own words: —

"If the patient was strong and plethoric, the pulse still full and distinct, the cramp severe, or there was great oppression in breathing, we would put the feet and legs in water as warm as could be borne, with the addition of mustard and common salt to the water; open a vein in the arm, and bleed from five, to sixteen or twenty ounces, watching the effect produced on the pulse and system generally; then place the patient in a warm bed, apply warmth to the feet and along the limbs; apply a large mustard cataplasm over the stomach, and give one of the pills of calomel, opium and camphor, every half hour. If the desire for cold drinks is not strong, the patient may drink from time to time a weak infusion of spear-mint, with the addition of eight or ten drops of camphorated spirits; if, however, the thirst is very intense, cold water may be used instead of the tea, and, in addition, small bits of ice given from time to time. The bed should be placed in the center of the room, without curtains, and the room should, if possible, be large, well ventilated, and if an upper room the better. If the weather will admit, the doors and windows should be kept open — the best stimulus for the patient is plenty of pure air — if the weather is damp, or chilly, so as to render it necessary to close the windows and doors, a little fire should be placed in an open fire-place, to temper the air; no persons should be admitted into the room except such as are necessary; every additional person renders the air more impure. The warm applications should be changed form time to time. If the patient be much exhausted or vomiting, on no consideration should he be permitted to get up to use the night vessel — the effort of getting up revives the vomiting, and this in its turn brings on the alvine discharges. If the patient be feeble and delicate, or if the discharges have been profuse, or if the fluids of the system have been drained off by long continuance of diarrhœa in the first stage, the bleeding must be omitted, and the other means adopted as before. If there is great precordial oppression, cups may be applied over the region of the stomach, and the mustard over the abdomen and over the stomach after the removal of the cups. If the pulse becomes gradually more full and distinct, and warmth returns to the surface, we have only to persevere in these means; after a time giving the pills less frequently, and permitting the patient to to take some light nourishment as chicken tea or light broth. If the perspiration becomes profuse — as will be likely — with a warm skin, it should be encouraged by taking some tepid aromatic drinks; the spearmint tea with a few drops of camphor is as good as any thing. If, notwithstanding these means, the patient continues to sink, or the profuseness of the discharges continue, we must resort to other means to try and arouse the system to reaction. Much care is, however, necessary to avoid throwing the patient into the fourth stage. Sulphuric ether in small doses should be given, and repeated every ten or fifteen minutes; or a week solution of carb. of ammonia may be substituted. At the same time an enema of a pint of chicken tea, with a table-spoonful of table salt, should from time to time be thrown into the bowels, and its retention secured for a few minutes by pressure on the fundament; a sheet must be placed under the patient to roceive the discharges, and on no account should be permitted to rise from the bed. If symptoms of reaction come on, the use of the stimulants should be gradually suspended. We believe that the more permanent and powerful stimulants, such as brandy, ardent spirits, &c. are only admissible where the patient has been addicted to their use. Too much precaution cannot be used during the period of convalescence as regards diet, exposure, &c. in all cases in which the disease has approached the stage of collapse."

The treatment of the *third* stage does not differ in its essential principles from that of the *second.* The grand object is the same, viz. "to arouse the dormant energies of the system by external warmth, pure air, and gentle and moderate stimulation." The author enjoins to continue persevering efforts under the most unpromising appearances, in as much as, although the prospect of recovery may really be small, there are no structural lesions which render recovery impossible, even when the symptoms are of the severest character.

The following quotation contains the plan to be pursued in the *fourth* stage:—

"If reaction be imperfectly established, the treatment must be governed by the variable condition of the patient. If the condition of the patient will admit, blood in moderate quantities may be drawn from the arm, but most frequently we shall be compelled to content ourselves with its abstraction by cupping and leeches, followed by fomentations and counter irritation. If the extremities are cold, warm and stimulating applications should be made to them. Cold may be applied to the head when painful; if the stomac is irritable, ice may be given, and the pills heretofore recommended, with the addition of one-fourth of a grain of ipecac to each pill. Mild nourishment should be given, and the pills continued until some impression is manifest on the general system. If typhoid symptoms supervene, it may be necessary to resort to tonics and stimulants, as sul. quinine, serpentaria, carb. of ammonia, wine whey, oil of turpentine, &c."

An *appendix* to this work contains the "Treatment of Epidemic Cholera by C. Searle, M. D. from the London Times;" "Report of a Committee of the Royal College of Physicians, London;" and "Report on Sanatary Measures, by the Board of Consulting Physicians of the City of Boston;" all interesting and valuable documents.

In concluding this brief and imperfect analytical sketch, we would again express the satisfaction which a perusal of this work has afforded us, and the hope that it will meet with an extensive circulation. It is a work to be commended in anticipation of the prevalence of the dreaded epedimic in different parts of our country, in as much as it is brief, and eminently practical. Nothing irrelevant and but little that is theoretical, have been admitted, to make a ponderous volume, discouraging to a practitioner, and impairing, by dilution, that which is useful. The aim of the author, as the work throughout bears internal evidence, has been to prepare a treatise which, at this juncture, shall be acceptable, and really valuable to the practitioner; ends which we doubt not, the readers of the work will regard as fulfilled.

We would also again advert to the fact that this is a Buffalo book, being one of several works that have lately been published by the enterprising book-sellers — G. H. Derby & Co. We trust the time may soon arrive when the publication of a valuable work in our flourishing city, will not admit of any comment on the score of the novelty of the event.

